# INNOVATING FOR EQUITABLE HOME HEALTH CARE: REDUCING BARRIERS TO SOCIAL AND END-OF-LIFE CARE FOR OLDER ADULTS

**DOI:** 10.1093/geroni/igae098.2057

**Published:** 2024-12-31

**Authors:** Komal Murali, Julia Burgdorf, Tracy Mroz

**Affiliations:** Chair; Co-Chair; Discussant

## Abstract

This symposium will focus on equity in home healthcare (HHC), operationalized as equitable access to, and receipt of, high quality HHC that is attuned to the unique needs of specific social and clinical subgroups, including minoritized racial/ethnic groups, those with chronic and serious illness such as dementia, and those at end-of-life. Topics include racial and ethnic disparities in hospice use and barriers to care, factors that impact individuals’ access to social work services, reasons for why palliative care is declined among individuals with lower levels of education, and disparities in access to high quality HHC for people living with dementia and their care partners. Abstracts present analyses from varied methodological and theoretical perspectives that all aim to better understand existing disparities and offer foundational evidence to support development of innovative models of care. The researchers and studies convened by this symposium are dedicated to equitable improvements in home health access and quality for the millions of diverse older adults who receive HHC each year in the United States. In sum, the robust findings of these impactful studies, each conducted in an interdisciplinary fashion and rooted in health-equity principles, present informative evidence for future work endeavoring to improve equitable access to and receipt of the full gamut of HHC services from early stages of illness and social care to end-of-life care. Hospice, Palliative and End-of-Life Care Interest Group Sponsored Symposium

